# The Role of the Microbiota in Esophageal Cancer

**DOI:** 10.3390/cancers15092576

**Published:** 2023-04-30

**Authors:** Clara Moreira, Ceu Figueiredo, Rui Manuel Ferreira

**Affiliations:** 1 Instituto de Investigação e Inovação em Saúde, Universidade do Porto (i3S), 4200-135 Porto, Portugal; 2 Department of Pathology, Faculty of Medicine of the University of Porto, 4200-319 Porto, Portugal; 3 Institute of Molecular Pathology and Immunology of the University of Porto (IPATIMUP), 4200-135 Porto, Portugal

**Keywords:** microbiota, esophageal cancer, esophageal squamous cell carcinoma, esophageal adenocarcinoma, dysbiosis, bacteria

## Abstract

**Simple Summary:**

Esophageal cancer has very high mortality and morbidity rates. In this study, we reviewed the current literature on the impact of microbiota on esophageal cancer and its precursor lesions. Globally, a decrease in microbiota richness and evenness in esophageal cancer is identified, which is accompanied by an increase in the abundance of Gram-negative bacteria.

**Abstract:**

Esophageal cancer is a major health problem, being the seventh most incidence cancer worldwide. Due to the often-late diagnosis and lack of efficient treatments, the overall 5-year survival is as low as 10%. Therefore, understanding the etiology and the mechanisms that drive the development of this type of cancer could improve the management of patients, increasing the chance of achieving a better clinical outcome. Recently, the microbiome has been studied as a putative etiological factor for esophageal cancer. Nevertheless, the number of studies tackling this issue is low, and the heterogeneity in the study design and data analysis has hindered consistent findings. In this work, we reviewed the current literature on the evaluation of the role of microbiota in the development of esophageal cancer. We analyzed the composition of the normal microbiota and the alterations found in precursor lesions, namely Barrett’s esophagus and dysplasia, as well as in esophageal cancer. Additionally, we explored how other environmental factors can modify microbiota and contribute to the development of this neoplasia. Finally, we identify critical aspects to be improved in future studies, with the aim of refining the interpretation of the relationship between the microbiome and esophageal cancer.

## 1. Introduction

The human microbiome comprises the whole set of microbial taxa and their genomes that inhabit different niches in the human body, including bacteria, fungi, and viruses. Usually, the term microbiota refers only to the collection of microorganisms themselves [1]. The number of bacteria colonizing the human body is frequently reported as outnumbering the number of host cells, but updated estimates propose a ratio of 1.3 bacterial cells for every human cell [2]. Although the number and abundance of bacteria are high, the diversity of the genomic information can be even greater since each species contains thousands of genes that contribute to a substantially more flexible and diverse metagenome than the human genome alone [3]. The microbiota plays important roles in the maintenance of human health, including metabolic activities, such as the production of vitamins and anti-inflammatory compounds, and also contributes contributing to the maturation of the immune system [4].

In recent years, the implementation of next-generation sequencing techniques has enabled in-depth characterization of the gastrointestinal microbiota, circumventing the limitations of the traditional culturing methods that only allowed the identification of cultivable species [4]. The study of the microbiota in a specific niche may involve the identification and quantification of each member of the community and the estimation of the diversity of the entire community, the latter varying from individual to individual and between groups of individuals. The composition of the microbial communities can be affected by diet, exercise, stress, aging, the intake of probiotics/prebiotics, antibiotics, or other drugs, as well as by the host immune system and host genetic factors [5]. Importantly, alterations in the equilibrium between the human host and the microbiota have been associated with the development of several diseases, namely cancer [6]. In several gastrointestinal cancers, including the stomach and the colon, dysbiosis has been consistently associated with cancer [6,7]. However, the mechanisms by which microbial dysbiosis can promote cancer are far from understood. One can speculate that the microbiota has direct oncogenic effects through the release of oncogenic toxins or secretion of oncometabolites, and/or indirect oncogenic actions, by the promotion and maintenance of local inflammation or by alteration of the systemic immune response.

Regarding esophageal cancer, the microbiota of the upper gastrointestinal tract has been suggested to play a role in the etiology of this malignancy, particularly in esophageal adenocarcinoma. Here, we review and discuss the literature that addresses the role of microbiota in the development of esophageal cancer. For this purpose, we conducted a search in the PubMed database for the query “esophageal cancer” AND “microbiota” in July 2022.

## 2. Esophageal Cancer

Esophageal cancer occupies the sixth position on the global ranking of cancer-related death, and it is the seventh most incident malignancy. This type of cancer was responsible for more than 544,000 deaths worldwide in 2020 [8]. Esophageal cancer incidence and mortality are unequally distributed around the world, being more incident and deadly in Eastern Asia and Southern Africa than in Western countries, such as France, Canada, and the USA [9].

The overall 5-year survival rate of esophageal cancer is approximately 10%, and the 5-year post-esophagectomy survival rate is between 15% to 40% [10]. Unfortunately, this disease is diagnosed at an advanced stage due to the lack of clinical symptoms, which contributes to the high mortality rate [10]. Since there are a small number of targeted therapies for esophageal cancer, the main treatment options are surgery, chemotherapy, and radiotherapy, as well as endoscopic therapies, such as radiofrequency ablation, endoscopic mucosal resection, and endoscopic submucosal dissection [11].

The great majority of esophageal cancers are esophageal squamous cell carcinomas (ESCC) or esophageal adenocarcinomas (EAC). ESCC is the most common type, representing 90% of esophageal cancers, having higher incidences in Asian and Eastern countries [12,13]. ESCC predominates in the upper and mid-thirds of the esophagus. It is thought to develop through the progression of premalignant precursor lesions of the squamous lining of the esophagus (squamous dysplasia), which result from the presence of chronic inflammation [14,15] that causes architectural changes such as disordered cellular stratification, loss of cellular polarity, and premature keratinization. Several risk factors have been associated with the development of ESCC, namely smoking, tobacco chewing, alcohol consumption, intake of hot beverages, and diets with reduced consumption of fresh fruits and vegetables [16,17,18].

At the molecular level, the ESCC subtype contains frequent mutations in *TP53, NFE2L2, MLL2, ZNF750, NOTCH1,* and *TGFBR2* genes and is characterized by upregulation of *Wnt*, syndecan, and p63 pathways, which are associated with the development and differentiation of squamous epithelial cells [19]. Epidermal growth factor receptor (EGFR) and receptor tyrosine kinase, or RAS signaling alterations, have been associated with ESCC and with a worse prognosis. In addition, vascular endothelial growth factor (VEGF) signaling pathway activation was also linked to ESCC and correlated with higher tumor stages and lymph node metastasis [10].

Although in Western countries, the incidence of EAC is typically low, EAC has been rapidly increasing, and in countries such as the United Kingdom, the Netherlands, the United States, Denmark, Canada, and Sweden, it has already overtaken ESCC [20,21]. The male sex, the Caucasian race, tobacco smoking, and obesity are the main risk factors for EAC. *Helicobacter pylori* infection in the stomach appears to be inversely associated with the incidence of EAC [22,23]. EAC occurs predominantly in the lower esophagus near the gastric junction and develops in the context of Barrett’s esophagus [24]. This condition in the esophagus arises from gastroesophageal reflux disease, which is a chronic digestive condition in which acidic contents from the stomach, frequently mixed with duodenal bile, enter the esophagus leading to esophageal tissue injury. At the cellular level, progression to EAC is underlined by continuous DNA damage caused by reflux and chronic inflammatory factors, which likely increase the mutation rate and promote genomic instability. *TP53* is the most frequently mutated gene in EAC tumors. A high frequency of mutations is also found in *CDKN2*, which is known to regulate the cell cycle [20]. These molecular alterations are consistent with the predominance of *CDKN2A* and *TP53* mutations in dysplastic Barrett’s esophagus, a precursor lesion of EAC. In addition, in EAC, mutations are frequently present in *ARID1A*, *SMAD4,* and *ERBB2* genes. Increased E-cadherin signaling and overexpression of *FOXA* and *ARF6,* which regulate E-cadherin during the endocytic pathway, may also be present in EAC [25].

## 3. Esophagus Microbiota in Healthy Conditions

The analysis of the bacterial community in the normal esophagus has been explored in a small number of studies (Table 1), probably due to difficulties in the recruitment of healthy volunteers for invasive sampling by upper endoscopy.

A Dutch study analyzed the mucosal microbiota composition along the entire gastrointestinal tract to find that the esophageal microbiota closely resembles the stomach microbiota rather than other communities in the gastrointestinal tract [26]. Proteobacteria were the most abundant phylum in the esophagus, followed by Firmicutes and Bacteroidetes [26]. Accordingly, the families Veillonellaceae, Streptococcaceae of the phylum Firmicutes, and Pseudomonadaceae of the phylum Proteobacteria were identified as the most abundant in the distal esophagus [26]. Subsequent studies that characterized the esophageal microbiota in biopsy specimens of American individuals and in mucosal brushings of Chinese subjects confirmed the high abundance of Firmicutes, followed by Proteobacteria, Bacteroidetes, Actinobacteria, and Fusobacteria [27,28,30]. Analysis of the esophageal mucosa microbiota of American individuals revealed that the top-5 most abundant genera were *Tissierella*, *Lactobacillus*, *Streptococcus*, *Acinetobacter*, and *Prevotella* [27]. *Streptococcus*, *Actinobacillus*, *Sphingomonas*, *Enterobacteriaceae*, *Neisseria*, *Haemophilus*, *Prevotella, Veillonella*, *and Porphyromonas* were identified as the most abundant members of the esophageal microbial community in a Chinese study that characterized the microbiota in brush specimens from 27 healthy individuals [28]. Of note, all the studies that profiled the microbiome at the genus level consistently identified the genus *Streptococcus* as one of the most abundant genera in the normal esophagus [28,29,30].

Despite the effort to characterize the normal esophagus microbiota, most publications included a low number of individuals and presented limitations regarding sequencing and data analysis sensitivity.

## 4. Modulators of the Esophageal Microbiota and of the Esophageal Cancer Risk

Several factors can disturb the esophageal microbiota, including alcohol consumption, dietary habits, smoking, obesity, and the intake of drugs, such as antibiotics or proton-pump inhibitors (PPIs) [31,32]. These elements are also important risk factors for the development of esophageal cancer, despite the fact that their association is restricted to specific cancer subtypes. For example, the intake of alcohol has been consistently associated with ESCC, but its association with EAC is unclear [33]. Regarding the esophageal microbiota, a recent study that analyzed 120 patients with ESCC, 60 of which were drinkers and 60 were non-drinkers, found that alcohol consumption was associated with alterations in the diversity and composition of the esophageal microbiota in ESCC patients. The microbiota of drinkers showed significantly lower alpha-diversity (within sample diversity) than that of non-drinkers, and their microbial profiles could be distinguished. Drinkers showed an enrichment of the order Pasteurellales, particularly the family Pasteurellaceae. In contrast, non-drinkers had a higher abundance of the class Clostridia, with specific enrichment of the bacterial families Clostridiaceae, Lanchnospiraceae, and Helicobacteraceae, and of the genera *Clostridium*, *Helicobacter*, and *Catonella* [34].

Diet can rapidly alter the structure and activity of the human gut microbiome [35]. Diets rich in lipids and sugars result in an increased body mass index and obesity, which are strongly associated with an increased risk of EAC [36]. Whether obesity modulates the human esophageal microbiota has not yet been addressed, but a study conducted in a mouse model suggests that high-fat diets influence the gut microbiota, contributing to the development of esophageal cancer [37]. Accordingly, the study reported that mice fed with a high-fat diet developed esophageal dysplasia and tumors more rapidly than those fed with a control diet. The high-fat diet resulted in an increased ratio of neutrophils to natural killer cells, which favors cancer development. Moreover, a high-fat diet led to a shift in the gut microbiota in comparison with a control diet. Analysis of microbial beta-diversity (between sample diversity) showed separation between mice fed with a high-fat diet and mice fed with the control diet. This differentiation was characterized by an altered ratio of Firmicutes to Bacteroidetes.

The exposure of fecal microbiota from mice fed the high-fat diet to mice fed the control diet accelerated tumor development in the esophagus. The relevance of the microbiota in the development of esophageal cancer was further demonstrated in animals raised in germ-free housing, which developed less dysplasia than mice grown under specific pathogen-free conditions [37]. In contrast to high-fat diets, the consumption of dietary fibers can modulate the microbial composition of the esophagus and have a protective role in esophageal neoplasia. A study comprising 47 patients demonstrated that fiber intake was positively associated with the relative abundance of Firmicutes [38]. In contrast, fiber intake was inversely associated with the relative abundance of Gram-negative Proteobacteria, which can be found in the abnormal esophagus, including reflux esophagitis and Barrett’s esophagus [39,40,41]. Altogether, these results are in line with those observed in the colon cancer model, where changes in the dietary content of fiber and fat have an impact on the colonic microbiome and metabolome, resulting in significant changes in mucosal inflammation and proliferation, thereby increasing colon cancer risk [42]. Smoking is a well-known risk factor for the development of cancer, but it can also shape the esophageal microbial communities through chemicals, heavy metals, and other constituents of tobacco [43]. A study of 278 male Chinese participants argued that current smokers tended to have increased microbial alpha-diversity compared to never smokers. The same study identified the enrichment of two anaerobes, *Dialister invisus,* and *Megasphaera micronuciformis,* in current smokers in comparison with never smokers [44].

PPIs can also modulate microbiota. These drugs are used to relieve symptoms caused by excessive acid production in the stomach, such as heartburn or gastroesophageal reflux disease. Despite anti-acid drugs targeting the acid-producing glands of the stomach, they can affect not only the gastric bacterial community but also the microbiota of the esophagus and the intestine [45]. It has been proposed that anti-acid drugs directly target the proton pumps containing P-type ATPase enzymes of specific bacteria, such as *Streptococcus pneumonia*, or indirectly increase the pH to levels that cause the death of certain bacterial species [46]. An investigation comparing the microbiota before and after PPI treatment in 16 patients with heartburn and normal-appearing mucosa and in 29 patients with abnormal mucosa due to esophagitis and Barrett’s esophagus found significantly different microbial profiles [47]. The treatment with PPIs was associated with increased abundance of the Comamonadaceae family and decreased abundance of the families Clostridiaceae, Lachnospiraceae, and two unclassified families of the orders Clostridia, Lactobacillales, and Gemelalles [47].

Antibiotics have been used to treat bacterial infections, but they can also disturb the resident bacteria. Despite this negative effect, in the context of cancer, specific antibiotics have been used to eradicate opportunistic microbes in cancer patients, resulting in potentially beneficial clinical outcomes [48]. Whether antibiotics have a protective effect against the development of esophageal cancer is poorly explored. In fact, one study using a rat mouse model showed a slight but non-significant reduction in the incidence of esophageal adenocarcinoma in animals treated with penicillin G and streptomycin in comparison with the control group [49]. This reduction was accompanied by an increased proportion of *Clostridium* clusters XIVa and XVIII and a decreased proportion of the *Lactobacillales* order [49].

## 5. Alterations in the Microbiota of Esophageal Premalignant Lesions

The role of the esophageal microbiota in the transition of normal epithelium to premalignant lesions and in the progression towards esophageal cancer is mostly unknown. So far, only a few studies have addressed this issue, and most of them presented limitations in sensitivity and depth of coverage, impairing the ability to draw clear and statistically based conclusions (Table 2). One of the first studies used an oral microbe identification microarray to characterize the presence of 272 microbial species in 142 Chinese patients with esophageal dysplasia and in 191 patients without esophageal dysplasia [50]. The study revealed an association between microbial richness and esophageal squamous dysplasia [50]. These results were confirmed in a more recent study that profiled the esophageal microbiota in 227 patients with normal esophageal function, low-grade dysplasia, high-grade dysplasia, and ESCC [30]. Disease progression was associated with decreased alpha-diversity and with a small but significant beta-diversity. Comparing the microbiota of dysplastic lesions with that of the normal esophagus, the genera *Atopobium*, *Enterococcus*, *Granulicatella*, *Lachnoanaerobaculum*, *Rothia*, Solobacterium, and *Streptococcus* were found to be enriched in low-grade dysplasia, while the genera *Bacillus*, *Lactobacillus* and *Streptococcus* were identified as markers of high-grade dysplasia [30].

Initial analyses of Barrett’s esophagus microbiota revealed that the esophageal microbiota could be categorized into two main types [40]. Type I was dominated by Gram-positive *Streptococcus* sp. and comprised cases with a morphologically normal esophagus. Conversely, type II was characterized by a high abundance of Gram-negative anaerobes and microaerophiles and was associated with esophagitis and Barrett’s esophagus [40]. Comparing the microbial community of Barrett’s esophagus with that of healthy controls, Lopetuso et al. identified only minor taxonomic differences, including the increase in the relative abundance of Fusobacteria at the phylum level and of *Leptotrichia* genus. However, this study noted a decrease in the abundance of the genera *Prevotella*, *Fusobacterium*, *Campylobacter*, and *Selenomonas* when metaplastic mucosa of Barrett’s esophagus was compared with adjacent normal areas of Barrett’s esophagus [55]. A larger case-control study reported marginal differences between the microbiota of Barrett’s esophagus and the microbial community of EAC. This study pointed to a significantly higher number of taxa but not to an overall difference in the microbial alpha-diversity of Barrett’s esophagus compared to that of EAC [24]. Moreover, Barrett’s lesions contained a significantly higher abundance of Proteobacteria than that found in EAC and in normal controls [24]. A study that combined 16S rRNA gene sequencing with whole metagenome sequencing identified 10 microbial taxa in gastroesophageal reflux disease patients in comparison with healthy controls, including *Prevotella intermedia*, *Prevotella micans*, *Neisseria macacae*, *Neisseria meningitidis*, *Haemophilus parainfluenzae*, and *Treponema medium*. The comparison of Barrett’s esophagus patients with controls revealed an increase in abundance of *Leptotrichia*, *Capnocytophaga*, *Gemella*, *Veillonella*, and *Streptococcus sanguinis*. The study pointed that distinct microbial community types are mainly defined by the relative abundance of the genera *Streptococcus* and *Prevotella* [53].

A study that aimed to reveal the potential role of the microbiota in the development of esophageal cancer profiled the microbial community of 45 patients, following the progression from Barrett’s esophagus to cancer [54]. The study reported a shift in the microbial composition of Barrett’s esophagus at the transition from low- to high-grade dysplasia. This was characterized by decreased abundance of Firmicutes, namely of *Veillonella* sp., an increased abundance of Proteobacteria of the Enterobacteriaceae family, and an increased proportion of samples with *Akkermansia muciniphila* in high-grade dysplasia.

## 6. The Esophageal Microbiota in Esophageal Cancer

Considering that esophageal cancer develops in the context of chronic inflammation, which is influenced by genetic and environmental factors, the esophageal microbiota is a possible etiological factor of esophageal cancer (Table 3).

In comparison with healthy controls, the microbial community of ESCC is characterized by reduced bacterial diversity [15,29,30] and by the enrichment of several genera, including *Streptococcus*, *Peptostreptococcus*, *Prevotella*, *Fusobacterium*, *Actinobacillus*, *Gemella*, *Rothia*, and Prevotella [15,29,30]. However, the analysis of the microbial community of tumor tissues and paired adjacent normal tissues did not identify consistent significant differences in the bacterial richness and diversity [56,57] but revealed a significant enrichment of *Fusobacterium* and *Streptococcus* and a decrease in the abundance of *Lactobacillus* in the ESCC tissue [57,58,59]. Interestingly, one study that included patients with ESCC and combined several genomics methods showed that the abundance of *Fusobacterium nucleatum* was significantly associated with the tumor tissue and not with the normal adjacent epithelium. This study pointed out that *F. nucleatum* was closely related to increased tumor staging and to the presence of mutations in genes such as *TP53*, *COL22A1*, *TRBV10*–1, *CSMD3*, *SCN7A,* and *PSG11* [57].

The microbial community of EAC is less characterized than ESCC. In fact, so far, only four studies evaluated the microbiota in EAC, with inconsistent results, probably due to differences in methodology, study design, and the limited number of cases included [24,27,54,55]. A study that performed a microbiome survey in only four patients with EAC, 25 with Barrett’s esophagus, and 16 healthy controls showed a decrease in the alpha-diversity in cases with non-dysplastic Barrett’s esophagus and EAC [54]. This study identified the enrichment of Enterobacteriaceae and *Akkermansia municiphila* and the decrease in *Veillonella* in cancer cases [54]. Another study that compared six EAC patients with 10 healthy controls identified a decrease in the microbial alpha-diversity in cancer in comparison with healthy controls, which was accompanied by a significant increase in the abundance of *Prevotella* sp., *Veillonella* sp., and *Leptotrichia* sp. [55]. On the other hand, Peter et al. identified in the EAC mucosa a significant decrease in the abundance of *Siphonobacter*, *Nitrosopumilus,* and *Planctomyces* in comparison tissue of healthy controls [27]. A larger study that compared the mucosal microbiota of 19 EAC with 20 healthy controls detected a significant increase in the abundance of *Streptococcus* and *Lactobacillus* in cancer cases [24]. This study suggested that *Lactobacillus* sp. dominate the niche of EAC due to their capability to resist low pH and to inhibit the growth of competitor bacteria through the production of lactic acid [24].

## 7. Non-Esophageal Microbiota and Esophageal Cancer

In recent years, several studies reported associations between the oral or the gut microbiota and increased risk of esophageal cancer. Given the invasive nature of sampling the esophagus through upper endoscopy for microbiome analysis, access to non-invasive oral or fecal specimens can be viewed as an opportunity to easily detect microbial biomarkers of esophageal cancer (Table 4).

Comparing the salivary bacterial community of 34 ESCC patients and 18 healthy controls from China, Chen et al. showed an overall decrease in alpha-diversity and clear separation of patient groups in beta-diversity matrices [6]. In accordance with these data, a Taiwanese study reported a decrease in bacterial richness in the saliva of patients with ESCC [6]. In contrast, two studies that also included Chinese patients did not find significant differences in the oral microbial alpha-diversity between ESCC and healthy controls [61,63]. When evaluating taxonomic differences, the relative abundance of *Prevotella*, *Streptococcus*, *Porphyromonas* [6], and *Neisseria* [63] was significantly higher in patients than in controls. In another study that examined the oral microbiome of 25 ESCC, 81 EAC patients, and 50 controls from the United States, Peters et al. did not find significant differences in the alpha-diversity [60]. The same study reported an association between the presence of *Porphyromonas gingivalis*, a pathogen associated with periodontal disease, and an increased risk of ESCC [60]. Moreover, the presence of the pathogen *Tannerella forsythia* and the enrichment of *Actinomyces cardiffensis*, *Selenomonas*, and *Veillonella* were significantly associated with an increased risk of EAC [60]. Zhao et al. compared the microbiota of 49 esophageal cancers with 51 healthy controls and detected higher abundances of *Prevotella nanceiensis*, *Neisseria weaveri*, and *Treponema vincentii* in cancer cases [63].

The relationship between the gut microbiome and esophageal cancer has been poorly explored [62,64]. In a case-control study that characterized the microbiome in fecal samples from 15 patients with esophageal cancer and 10 healthy controls, significant alterations in the microbiota were identified, which are compatible with gut microbial dysbiosis in cancer patients [62]. This study detected increased diversity in the fecal microbiota of cancer patients in comparison with that of healthy controls. Moreover, significant enrichment in the abundances of Bacteroidetes, Bacteroidaceae, Enterobacteriaceae, and of *Escherichia-Shigella*, was identified in esophageal cancer patients [62]. In another study that included 23 esophageal cancers and 23 healthy controls, a significant increase in microbial richness was detected in the gut microbiota of cancer patients [64]. Analysis of the microbial profile by hierarchical clustering revealed a clear separation of the microbial community between esophageal cancer patients and controls. These differences were mainly attributed to the enrichment in cancer patients of several genera, including *Streptococcus*, *Bifidobacterium*, *Subdoligranulum*, *Blautia*, *Romboutsia*, *Collinsella*, *Paeniclostridium*, *Dorea*, and *Atopobium* [64].

## 8. Conclusions and Future Directions

Esophageal cancer is a very relevant pathology both in terms of mortality and morbidity. It is now accepted that the esophageal microbiome is altered in esophageal carcinogenesis. 

Some of the classical risk factors associated with esophageal cancer appear to be associated with changes in the normal microbiota of the esophagus. Alcohol consumption has been linked with alterations of the diversity of the microbiota in ESCC patients. Diets rich in high-fat contents in animal studies have been linked with esophageal dysplasia and alterations of the microbiota. The low intake of fibers was also associated with an increase in Gram-negative species, which can be found in esophageal cancer precursor lesions. Smoking, and drugs such as PPIs and antibiotics, have been associated to changes in the esophageal bacterial population. Overall, the microbiota of the normal esophagus is mainly composed of Firmicutes, Proteobacteria, Bacteriodetes, Actinobacteria, and Fusobacteria. In contrast, esophageal cancer is characterized by reduced microbial diversity, which appears to begin in the precursor stages of esophageal cancer. At the taxonomic level, the esophageal cancer microbiota is characterized by a shift from Gram-positive to Gram-negative bacteria. The genera most commonly enriched in esophageal cancer are *Fusobacterium*, *Streptococcus*, *Veilonella*, and *Prevotella*. 

Even though one can pinpoint specific alterations in the esophageal cancer microbiota, a consistent esophageal cancer-associated microbiota profile has not yet been identified. This lack of consistency can be due to the different technical approaches, such as the sampling method, the type of samples analyzed, and the analytic strategy used to profile the microbiome, as well as to the relatively small number of individuals included in each study. Future studies should consider the standardization of the methods, namely DNA isolation, 16S rRNA amplification and sequencing, and data analysis, which will positively impact the reliability of the results. In addition, the inclusion of blanks to control potential microbial contaminations, which can arise during sample processing, will improve the quality of the final datasets. The use of molecular approaches alternative to 16S rRNA gene sequencing, such as whole metagenome sequencing, may help disclose in greater detail the taxonomic profile to the species level and also allow the evaluation of the functional content of the microbiome. Although these methods have never been applied to the mucosal esophageal microbiome, they have the potential to identify microbial toxins, virulence factors, and enzymes that can influence carcinogenesis in the esophagus.

Additional relevant aspects need to be considered when addressing the relationship between the microbiome and esophageal cancer. The inclusion of patients of distinct geographic origins will be important to control the influences of the host genetic diversity and distinct cultural lifestyles. Furthermore, the collection of data on dietary habits, use of medication, and lifestyle factors of each individual will be relevant to control potential confounding variables in the observed microbiota profiles. In future studies in the setting of esophageal carcinogenesis associated with the microbiome, multidisciplinary research efforts will be fundamental to analyze and integrate the effects of multiple exposures, including those of the diet, with the microbiome and with the changes that occur along the process of carcinogenesis [66]. The dissection of such complex interactions would benefit from large and well-characterized prospective cohort studies and would certainly contribute to designing novel prevention and treatment strategies to reduce the burden of esophageal cancer.

## Figures and Tables

**Table 1:** Summary of the studies evaluating the esophageal microbiota in healthy individuals.

Reference	Country	No. Participants	Specimen and Measurement	Taxonomic Findings in Healthy Individuals *
Vuik et al. 2019 [26]	The Netherlands	HC (13) RE (1)	Biopsy specimens 16S rRNA gene sequencing	Veillonellacea _(F)_ Psudomonadaceae _(F)_ Streptoccaceae _(F)_
Peter et al. 2020 [27]	USA	HC (10) IM (10) HGD (10) LGD (10) EAC (12)	Biopsy specimens 16S rRNA gene sequencing	At the phylum-level: Firmicutes Proteobacteria Bacteriodetes Actinobacteria Fusobacteria At the genus-level: *Tissierella* *Streptococcus* *Lactobacillus* *Acinetobacter* *Prevotela* *Fusobacterium* *Staphylococcus* *Akkermansia* *Blautia*
Yin et al. 2020 [28]	China	HC (27)	Esophageal brush specimens 16S rRNA gene sequencing	At the phylum-level: Proteobacteria Firmicutes Bacteroidetes Actinobacteria Fusobacteria TM7 At the genus-level: *Streptococcus* *Actinobacillus* *Sphingomonas* unclassified Enterobacteriaceae *Neisseria* *Haemophilus* *Prevotella* *Veillonella* *Porphyromonas*
Li et al. 2020 [29]	China	HC (16) ESCC (17) EGJ (11)	Esophageal brush specimens 16S rRNA gene sequencing	At the phylum-level: Proteobacteria Firmicutes Bacteroidetes Actinobacteria At the genus-level: *Streptococcus* *Ralstonia* *Burkholderia–Caballeronia–Paraburkholderia* *Fusobacterium*
Li et al. 2021 [30]	China	HC (82) LGD (60) HGD (64) ESCC (70)	Esophageal brush specimens 16S rRNA gene sequencing	At the phylum-level: Firmicutes Proteobacteria Bacteroidetes Actinobacteria Fusobacteria At the genus-level: *Streptococcus* *Neisseria* *Haemophilus* *Prevotella*

EAC—esophageal adenocarcinoma; EGJ—esophagogastric junction cancer, F—family; HC—Healthy controls; HGD—high-grade dysplasia; IM—Intestinal metaplasia, LGD—low-grade dysplasia; RE—reflux esophagitis. * When data are available, taxa are displayed in descending order of abundance.

**Table 2:** Studies characterizing the esophageal microbiota in esophageal premalignant lesions.

Reference	Country (N)	Specimen and Measurement	Diversity		Taxonomic Findings
			Alpha	Beta	
Macfarlane et al. 2007 [51]	USA No BE (7) BE (7)	Biopsy; Aspirate 16S rRNA gene sequencing	↑ BE	NS	↑ *Veillonella* _(G)_ ↑ *Neisseria* _(G)_ ↑ *Camplylobacter* _(G)_ ↑ *Fusobacterium* _(G)_ ↑ *Megasphaera* _(G)_ ↑ *Staphylococcus* _(G)_ ↓ *Lactobacillus* _(G)_
Yang et al. 2009 [40]	USA HC (12) ES (12) BE (10)	Biopsy 16S rRNA gene sequencing	↑ Type II compared to type I	Distinguished Type I and Type II microbiome	Type I microbiome: Normal esophagus Type II microbiome: Abnormal esophagus Type II vs. Type I: ↑ Gram-negative bacteria ↑ *Veillonella*_(G)_ ↑ *Prevotella* _(G)_ ↑ *Haemophilus _(G)_* ↑ *Neisseria* _(G)_ ↑ *Rothia* _(G)_ ↑ *Granulicatella* _(G)_ ↑ *Campylobacter* _(G)_ ↑ *Porphyromonas* _(G)_ ↑ *Fusobacterium _(G)_* ↑ *Actinomyces _(G)_* ↓ Firmicutes _(P)_ ↓ *Streptoccocus* _(G)_
Liu et al. 2013 [52]	Japan HC (6) ES (6) BE (6)	Biopsy 16S rRNA gene sequencing	NS	NS	ES/HC vs. BE: ↓ *Streptococcus* _(G)_ ES/BE vs. HC: ↑ *Veillonella* _(G)_ ↑ *Neisseria* _(G)_ ↑ *Fusobacterium* _(G)_
Yu et al. 2014 [50]	China No ESD (191) ESD (142)	Brush Human oral microbe identification microarray	↓ ESD	Distinguished ESD and No ESD	Inverse association between microbial richness and ESD when comparing with non ESD
Elliot et al. 2017 [24]	UK HC (20) NDBE (24) BE (23) EAC (19)	Biopsy, Brushing, Cytosponge 16S rRNA gene sequencing	NS	Distinguished HC and BE	↑ Proteobacteria _(P)_
Deshpande et al. 2018 [53]	Australia HC (59) GERD (29) GM (7) BE (5) EAC (1) EoE (1)	Esophageal brush specimens 16S rRNA gene sequencing Whole metagenome sequencing	NS	NS	GERD vs. HC: ↑ Flavobacteriaceae _(F)_ ↑ *Acetoanaerobium* _(G)_ ↑ *Filifactor* _(G)_ ↑ *Campylobacter* _(G)_ ↑ *Prevotella intermedia* _(S)_ ↑ *Prevotella micans* _(S)_ ↑ *Neisseria macacae* _(S)_ ↑ *Neisseria meningitidis*_(S)_ ↑ *Haemophilus parainfluenzae* _(S)_ ↑ *Treponema medium* _(S)_ BE vs. HC: ↑ *Leptotrichia* _(G)_ ↑ *Capnocytophaga* _(G)_ ↑ *Gemella*_(G)_ ↑ *Veillonella*_(G)_ ↑ *Streptococcus sanguinis*_(S)_
Snider et al. 2019 [54]	USA HC (16) NDBE (14) LGD (6) HGD (5) EAC (4)	Brush 16S rRNA gene sequencing	NS	NS	HGD vs. LGD: ↑ Proteobacteria _(P)_ ↓ Firmicutes _(P)_ ↓ *Veillonella* _(G)_
Lopetuso et al. 2020 [55]	Italy HC (10) BE (10) BEU (10) EAC (6)	Biopsy 16S rRNA gene sequencing	↓ BE	Distinguished HC and BE	BE vs. HC: ↑ Fusobacteria _(P)_ ↑ *Leptotrichia* _(G)_ BE vs. BEU: ↓ Bacteroidetes _(P)_ ↓ TM7 _(P)_ ↓ Prevotellaceae _(F)_ ↓ Veillonellaceae _(F)_ ↓ Fusobacteriaceae _(F)_ ↓ Lachnospiraceae _(F)_ ↓ Campylobacteraceae _(F)_ ↓ *Prevotella* _(G)_ ↓ *Fusobacterium* _(G)_ ↓ *Campylobacter* _(G)_ ↓ *Selenomonas* _(G)_
Li et al. 2021 [30]	China HC (82) LGD (60) HGD (64) ESCC (70)	Brush 16S rRNA gene sequencing	↓ HGD compared to HC/LGD	Distinguished HC/LGD group and HGD	LGD vs. HC: ↑ *Atopobium* _(G)_ ↑ *Enterococcus* _(G)_ ↑ *Granulicatella* _(G)_ ↑ *Lachnnoanaerobaculum* _(G)_ ↑ *Rothia* _(G)_ ↑ *Solobacterium* _(G)_ ↑ *Streptococcus* _(G)_ HGD vs. HC: ↑ *Bacillus* _(G)_ ↑ *Lactobacillus* _(G)_ ↑ *Streptococcus* _(G)_

AHT—adjacent mucosa healthy tissue; BE—Barrett’s esophagus; BEU—unaffected esophageal mucosa of Barrett’s esophagus patient; EAC—esophageal adenocarcinoma; ES—esophagitis; ESD—esophageal squamous dysplasia; GERD—gastroesophageal reflux disease; GM—glandular mucosa; EoE—Eosinophilic esophagitis; HC—healthy control, HGD—High grade dysplasia; LGD—Low grade dysplasia, NDBE—non-dysplastic Barrett’s esophagus; NS—not significant; P—phylum; F—family; G—genus; S—species; ↑—increase; ↓—decrease.

**Table 3:** Summary of the studies evaluating esophageal microbiota in esophageal cancer.

Reference	Country (N)	Specimen and Measurement	Diversity		Taxonomic Findings
			Alpha	Beta	
Elliott et al. 2017 [24] *	UK HC (20) NDBE (24) BE (23) EAC (19)	Biopsy, Brushing, Cytosponge 16S rRNA gene sequencing	↓ EAC in comparison with HC	Distinguished EAC and HC	EAC vs. HC: ↑ Lactobacillales _(O)_ ↑ Coriobacteriacea _(F)_ ↑ Lactobacillaceae _(F)_ ↑ *Streptococcus* _(G)_ ↑ *Lactobacillus* _(G)_
Shao et al. 2019 [56] **	China ESCC (67) NAM (67)	Biopsy 16S rRNA gene sequencing	NS	Distinguished ESCC and NAM	ESCC vs. NAM: ↑ Fusobacteria _(P)_ ↓ Firmicutes _(P)_ ↓ *Fusobacterium* _(G)_ ↓ *Streptococcus* _(G)_
Snider et al. 2019 [54] *	USA HC (16) NDBE (14) LGD (6) HGD (5) EAC (4)	Brush 16S rRNA gene sequencing	↓ EAC in comparison with NDBE, LGD and HGD	NS	HGD/EAC vs. NDBE/LGD: ↓ Firmicutes _(P)_ ↑ *Proteobacteria* _(P)_ ↑ Enterobacteriacea _(F)_ ↑ *Akkermansia muciniphila* _(S)_ ↓ *Veillonella* _(G)_
Lopetuso et al. 2020 [55] *	Italy HC (10) BE (10) EAC (6)	Biopsy 16S rRNA gene sequencing	↑ EAC in comparison with HC	Distinguished EAC and HC	EAC vs. HC: ↑ *Prevotella* _(G)_ ↑ *Leptotrichia* _(G)_ ↑ *Veillonella* _(G)_ ↑ *Bacteroidetes* _(G)_ ↓ *Streptococcus* _(G)_ ↓ *Granulicatella* _(G)_
Li et al. 2020 [29] *	China HC (16) RE (15) EGJ (11) ESCC (17)	Brush 16S rRNA gene sequencing	↓ ESCC and EGJ in comparison with HC	Distinguished ESCC and HC	ESCC vs. HC: ↑ Fusobacteria _(P)_ ↓ Actinobacteria _(P)_
Peter et al. 2020 [27] *	USA IM (10) LGD (10) HGD (10) EAC (12) HC (10)	Biopsy 16S rRNA gene sequencing	NS	NS	EAC vs. HC: ↓ Planctomycetes _(P)_ ↓ Crenachaeota _(P)_ ↓ *Siphonobacter* _(G)_ ↓ *Nitrosopumilus* _(G)_ ↓ *Planctomyces* _(G)_
Li et al. 2021 [30] *	China HC (82) LGD (60) HGD (64) ESCC (70)	Brush 16S rRNA gene sequencing	↓ ESCC in comparison with HC	Distinguished ESCC and HC	ESCC vs. HC: ↑ *Peptoniphilus* _(G)_ ↑ *Petostreptococcus* _(G)_ ↑ *Lachnospiraceae_[G9]*_(G)_ ↑ *Bosea* _(G)_ ↑ *Gemella* _(G)_ ↑ *Solabacterium* _(G)_ ↑ *Streptococcus* _(G)_
Li et al. 2021 [57] **	China ESCC (41) NAM (41)	Biopsy 16S rRNA gene sequencing	↓ ESCC in comparison with NAM	Distinguished ESCC and NAM	ESCC vs. NAM: ↑ Bacteroidetes _(P)_ ↑ Fusobacteria _(P)_ ↑ Spirochaetae _(P)_ ↓ Proteobacteria _(P)_ ↓ Firmicutes _(P)_ ↓ Actinobacteria _(P)_ ↑ *Streptococcus* _(G)_ ↑ *Fusobacterium* _(G)_ ↑ *Prevotella* _(G)_ ↓ *Butyrivibrio* _(G)_ ↓ *Lactobacillus* _(G)_
Jiang et al. 2021 [15] *	China HC (21) ES (15) ESCC (32)	Biopsy 16S rRNA gene sequencing	↑ ESCC in comparison with HC	Distinguished ESCC and HC	ESCC vs. HC: ↓ Fusobacteria _(P)_ ↑ *Streptococcus* _(G)_ ↑ *Actinobacillus* _(G)_ ↑ *Peptostreptococcus* _(G)_ ↑ *Fusobacterium* _(G)_ ↑ *Prevotella* _(G)_ ↓ *Bacteroides* _(G)_ ↓ *Faecalibacterium* _(G)_ ↓ *Curvibacter* _(G)_ ↓ *Blautia* _(G)_
Shen 2022 et al. [58] **	China ESCC (51) NAM (51)	Biopsy 16S rRNA gene sequencing Species-specific qPCR	NS	NS	ESCC vs. NAM: ↓ Deinococcus-Thermus _(P)_ ↑ Spirochaetes _(P)_ ↑ Tenericutes _(P)_ ↓ Actinobacteria _(P)_ ↓ Verrumicrobia _(P)_ ↑ Lentimicrobiaceae _(F)_ ↑ *Treponema* _(G)_ ↑ *Selenomonas* _(G)_ ↑ *Peptonaerobacter* _(G)_ ↓ *Methylobacter* _(G)_ ↓ *Akkermansia* _(G)_ ↑ *Blautia* _(G)_ ↑ *Labrys ginsengisoli*_(S)_ ↑ *Peptoanaerobacter stomatis* _(S)_ ↑ *Selenomonas sputigena* _(S)_ ↑ *Streptococcus constellatus*_(S)_ ↑ *Fusobacterium periodonticum* _(S)_ ↓ *Lactobacillus murinus* _(S)_ ↓ *Escherichia coli* _(S)_

* Studies comparing esophageal cancer with healthy subjects. ** Studies comparing paired normal adjacent mucosa with tumor tissue. BE—Barrett’s esophagus; C—class; EAC—esophageal adenocarcinoma; EC—esophageal cancer; EGJ—esophagogastric junction cancer; ES—esophagitis; ESCC—esophageal squamous cell carcinoma; ESD—esophageal squamous dysplasia; F—family; G—genus; HC—healthy controls; HGD—high grade dysplasia; IM—intestinal metaplasia; LGD—low grade dysplasia; MEE—mid-esophageal esophagitis; NAM—normal adjacent mucosa; NDBE—non-dysplastic Barrett’s esophagus; NS—no significant difference; RE—after radical esophagectomy; P—phylum; O—order; F—family; S—species; ↑—increase; ↓—decrease.

**Table 4:** Studies show relationships between non-esophageal microbiota and esophageal cancer.

Reference	Country (N)	Specimen and Measurement	Diversity		Taxonomic Findings
			Alpha	Beta	
Peters et al. 2017 [60]	USA HC (210) ESCC (25) EAC (81)	Mouthwash 16S rRNA gene sequencing	NS	NS	EAC: ↑ *Selenomonas* _(G)_ ↑ *Veillonella* _(G)_ ↑ *Tannerella forsythia* _(S)_ ↑ *Actinomyces cardiffensis* _(S)_ ESCC: ↑ *Bergeyella* _(G)_ ↑ *Porphyromonas gingivales* _(S)_ ↑ *Prevotella nanceiensis* _(S)_ ↑ *Neisseria weaver* _(S)_ ↑ *Treponema vincentii* _(S)_
Wang et al. 2019 [61]	China HC (21) ESCC (20)	Saliva 16S rRNA gene sequencing	NS	Distinguished HC and ESCC	ESCC vs. HC: ↑ Firmicutes _(P)_ ↓ Gammaproteobacteria _(C)_ ↑ *Bacillus* _(G)_ ↑ *Lactobacillus* _(G)_
Ishaq et al. 2021 [62]	China HC (10) EC (15)	Biopsy DGGE 16S rRNA gene sequencing	↑ EC	Distinguished HC and EC	EC vs. HC: ↑ Bacteroidetes _(P)_ ↑ Bacteroidaceae _(F)_ ↑ Enterobacteriaceae _(F)_ ↑ *Bacteroides* _(G)_ ↑ *Escherichia*-*Shigella* _(G)_ ↓ Prevotellaceae _(F)_ ↓ Veillonellaceae _(F)_ ↓ *Prevotella* _(G)_ ↓ *Dialister* _(G)_
Chen et al. 2021 [6]	Taiwan HC (18) ESCC (34)	Oral biofilm; Biopsy *P. gingivalis* qPCR; 16S rRNA gene sequencing	↓ ESCC	Distinguished HC and ESCC	ESCC vs. HC: Oral biofilm: ↑ *Streptococcus* _(G)_ ↑ *Veillonella* _(G)_ ↑ *Porphyromonas gingivalis* _(S)_ Biopsy: ↑ *P. gingivalis* _(S)_
Zhao et al. 2020 [63]	China HC (51) EC (49)	Saliva 16S rRNA gene sequencing	NS	Distinguished HC and EC	EC vs. HC: ↑ Firmicutes _(P)_ ↓ Proteobacteria _(P)_ ↑ Negativicutes _(C)_ ↓ Betaproteobacteria _(C)_ ↑ Selenomonadales _(O)_ ↓ Neisseriales _(O)_ ↑ Veillonellaceae _(F)_ ↑ Prevotellaceae _(F)_ ↓ Neisseriaceae _(F)_ ↑ *Prevotella* _(G)_ ↓ *Neisseria* _(G)_
Deng et al. 2021 [64]	China HC (23) EC (23)	Stool 16S rRNA gene sequencing	↑ richness in EC	Distinguished HC and EC	EC vs. HC: ↓ Bacteroides _(P)_ ↑ *Streptococcus* _(G)_ ↑ *Bifidobacterium* _(G)_ ↑ *Subdoligranulum* _(G)_ ↑ *Blautia* _(G)_ ↑ *Romboutsia* _(G)_ ↑ *Collinsella* _(G)_ ↑ *Paeniclostridium* _(G)_ ↑ *Dorea* _(G)_ ↑ *Atopobium* _(G)_ ↓ *Lachnospira* _(G)_ ↓ *Bacteroides* _(G)_ ↓ *Agathobacter* _(G)_ ↓ *Lachnoclostridium* _(G)_ ↓ *Parabacteroides* _(G)_ ↓ *Paraprevotella* _(G)_ ↓ *Butyricicoccus* _(G)_ ↓ *Tyzzerella* _(G)_ ↓ *Fusicatenibacter* _(G)_ ↓ *Sutterella* _(G)_
Wu et al. 2022 [65]	China HC (40) EC (40)	Stool Culture	–	–	EC vs. HC: ↑ *Enterococcus* _(G)_ ↑ *Escherichia coli* _(S)_ ↓ *Bifidobacterium* _(G)_ ↓ *Lactobacillus* _(G)_

C—class; DS—dysplasia; EAC—esophageal adenocarcinoma; EC—esophageal cancer; ESCC—esophageal squamous cell carcinoma; F—family; G—genus; NS—no significant difference; O—order; P—phylum, ↑—increase; ↓—decrease.
